# Esophageal atresia with tracheoesophageal fistula: two case reports

**DOI:** 10.1186/s13256-023-04278-1

**Published:** 2023-12-31

**Authors:** Naomi A. Mwamanenge, Haika K. Mariki, Lucy L. Mpayo, Ernestina E. Mwipopo, Fatima Mussa, Masawa K. Nyamuryekung’e, Yahaya Binde, Said Kiangi, Zaituni Bokhary, Martha Mkony, Yaser Abdallah, Karim Manji

**Affiliations:** 1 Muhimbili University of Health and Allied Sciences (Muhas), P. O box 65001, Dar es Salaam, Tanzania; 2 Muhimbili National Hospital (Mnh), Dar es Salaam, Tanzania; 3 Aga Khan Hospital, Dar es Salaam, Tanzania

**Keywords:** Esophageal atresia, Tracheoesophageal fistula, Pneumothorax, Anastomosis leak, Sepsis

## Abstract

**Background:**

The incidence of esophageal atresia with tracheoesophageal fistula is 1 out of 3000–5000 live births. Its incidence in lower middle income countries is not known. The infants usually present with excessive secretions or choking while feeding and are at risk for aspiration. The outcome of these infants in lower middle income countries is not encouraging due to delays in referral, sepsis at presentation requiring preoperative stabilization, postoperative complications such as anastomosis leaks, pneumonia, and pneumothorax.

**Case presentation:**

We present two African babies who were term infants at age 2 days (male) and 5 days (female) with diagnosis of esophageal atresia and tracheoesophageal fistula. The 5-day-old infant required preoperative stabilization due to sepsis and delayed surgery with a poor postoperative outcome. The 2-day-old infant was preoperatively stable and had a good postoperative outcome. The challenges faced in management of these two cases have been highlighted.

**Conclusion:**

Outcome of infants with esophageal atresia and tracheoesophageal fistula in lower middle income countries is not encouraging due to delays in referral and poor postoperative healing attributed to sepsis and recurrent pneumothorax. Timely referral, preoperative condition of the infant, and timely management has shown to be a contributory factor for an improved outcome.

## Introduction

The incidence of esophageal atresia (EA) with tracheoesophageal fistula is estimated to be 1 out of 3000–5000 live births [1, 2]; however, the incidence in low income countries is not well known. Tracheoesophageal fistula (TEF) is thought to occur during embryogenesis at the 4th week of gestational age (GA) when separation of the primitive trachea and esophagus are thought to take place [3]. Due to delays in diagnosis and referral, the management and prognosis of these infants can become poor [4]. In lower middle income countries such as Tanzania, antenatal diagnosis is a challenge, and when postnatal diagnosis is made, there are delays in referral to the tertiary center. As a result, a majority of these infants come when they are critically ill, and the postoperative healing process becomes a challenge due to sepsis and other complications such as pneumothorax or anastomosis leak [5]. We have had a couple of infants with TEF and EA who were referred for further management in our neonatal unit; unfortunately, their outcome was not encouraging, except for only one who survived. The reasons for poor outcome of these infants were due to delay referral causing infants to arrive in critical condition and, thereafter, a delay in surgery due to initial stabilization. Once surgery was done, there was poor postoperative healing due to sepsis, anastomosis leaks, pneumonia, and postoperative pneumothorax. We report two cases among these, to share experiences and highlight challenges in care of TEF/EA in lower middle income countries such as Tanzania.

## Case 1: the neonate who did not survive

A 5-day-old term, African female baby was referred from the central region, almost 600 km away from Dar es Salaam, due to difficulties in breathing and excessive secretion in the mouth; she was choking while breastfeeding with a lot of milk coming out through the nose. An attempt to insert a nasogastric tube (NGT) was not successful.

Both parents are in their early twenties and do not have any diseases. This was their second child, while the other sibling is well. No significant family history forthcoming and no history of consanguinity were seen. She was diagnosed negative for venereal disease research laboratory (VDRL), human immunodeficiency virus (HIV), and hepatitis B tests. There were no history of pregnancy-induced hypertension or gestational diabetes throughout the pregnancy. Her antenatal ultrasound done at 24 weeks GA was normal.

The infant was delivered normally and weighed 2800 g with an Apgar score 8 and 9 in the 1st and 5th minute, respectively. Upon arrival at the neonatal intensive care unit (NICU), she had respiratory distress and excessive secretions in the mouth. She was afebrile, had good neonatal reflexes, and had no external dysmorphic features. Respiratory examination revealed a respiratory rate of 80 beats/minute, and auscultation revealed bilateral crepitation. Cardiovascular examination revealed a grade 3 machinery murmur at upper left sternal border. Examination of the abdomen was normal and anal opening was patent.

**Fig. 1:**
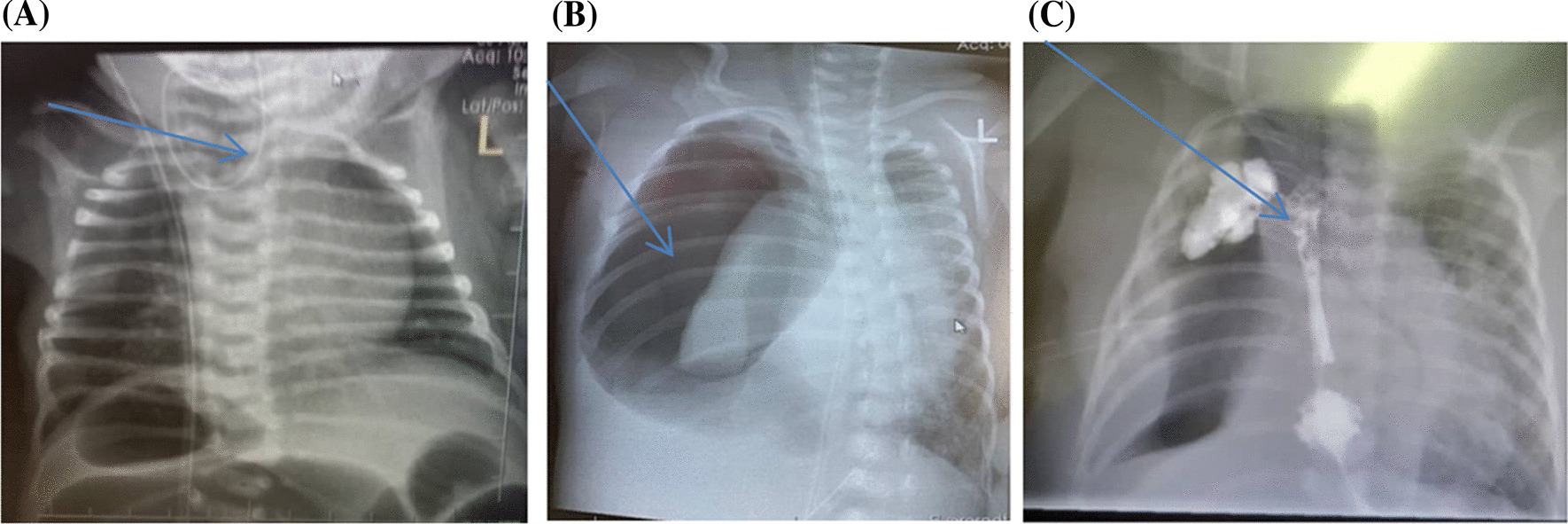
**A** Chest X-ray showing coiling of nasogastric tube, blue arrow shows coiling of nasogastric tube in upper esophageal pouch. **B** Chest X-ray showing pneumothorax, blue arrow shows right tension pneumothorax, collapse of ipsilateral lung and contalateral shift of the mediastinum. **C** Chest X-ray showing anastomotic leakage, blue arrtow shows leakage of the gastrografin swallow into the right lung

The chest X-ray (Fig. 1A) showed recoiling of the nasogastric tube and presence of gastric bubbles suggestive of a type C TEF and features of aspiration pneumonia. Echocardiogram showed a moderate patent ductus arteriosus (PDA) measuring 2.5 mm and a dilated left heart. Complete blood count (CBC) and C-reactive protein (CRP) results were normal. Ceftriaxone was initiated for the aspiration pneumonia. Total parenteral nutrition (TPN) was given and continuous suction of the oral secretion was done.

On the 13th day of life (9 days after admission), the baby was operated on. A right posterolateral thoracotomy incision was done, and the intraoperative findings were a blind-end proximal loop of the esophagus with the distal loop having a fistula to the trachea. End-to-end anastomosis of esophagus was done and the fistula was ligated. Postoperatively, she continued to be ventilated but, a few hours later, started to have increased requirements of oxygen and ventilator support. The chest was bulging and tympanic, and emergency portable X-ray revealed a massive tension pneumothorax on the right side with mediastinal shift to the left (Fig. 1B). Decompression was done and a chest tube drain was inserted and left *in situ*.

The baby had shown improvement, however on the 10th postoperative day (23rd day of life), there were significant mucoid, serosanguineous secretions via the chest tube. She progressively became sicker, lethargic, and pale. Laboratory investigations were done including CBC, CRP, and blood culture. Antibiotics were changed to meropenem due to a raised CRP, pending blood culture results. Blood culture revealed *Serratia marcesnus* sensitive to piperacin and tazobactam. A blood transfusion was given due to low hemoglobin of 7 g/dl. TPN continued.

The baby continued to deteriorate. Gastrografin swallow showed anastomosis leakage (Fig. 1C). She developed another tension pneumothorax, and decompression was done again. However, despite all the efforts, the baby succumbed at day of life 34 (21 days postoperative).

## Case 2: the surviving neonate

A 2-day-old term, African male baby was delivered via caesarean section (CS) due to previous CS of the mother. The baby was referred to our hospital from one of the hospitals in Dar es Salaam for pediatric surgical expertise. The birth weight was 2400 g with an Apgar score of 9 and 10 at the 1st and 5th minute, respectively.

The mother is in her early thirties and this is her second child. She attended a total of four antenatal visits and received her vaccines for tetanus toxoid. She was dewormed and given malaria prophylaxis. Serology was negative for VDRL, HIV, and hepatitis B. She was normotensive and random sugar had been within normal throughout the pregnancy. Her antenatal hemoglobin (Hb) ranged 11.5 to 12 g/dl, and a day before delivery it was 11.7 g/dl. Antenatal ultrasound done at 22 weeks of GA was normal, with no report of oligohydramnions/polyhydramnions or anomalies detected via scan.

The baby was admitted due to history of difficulties in breathing since birth, excessive frothing, and choking in an attempt to breastfeed. Esophageal atresia was suspected, and an attempt to pass a nasogastric tube was not successful. General examination of the baby revealed an absence of anal opening. Repair of the anal malformation with subsequent double bowel colostomy placement was done at the referring hospital. The baby was then transferred to a tertiary care facility with pediatric surgery capabilities for repair of the fistula.

Upon arrival at our facility, the baby had respiratory distress with frothing from the oral cavity. Continuous suctioning of the blind esophageal pouch, oxygen supplementation, total parental nutrition (TPN), and intravenous antibiotic ceftriaxone were administered as per requirement.

**Fig. 2:**
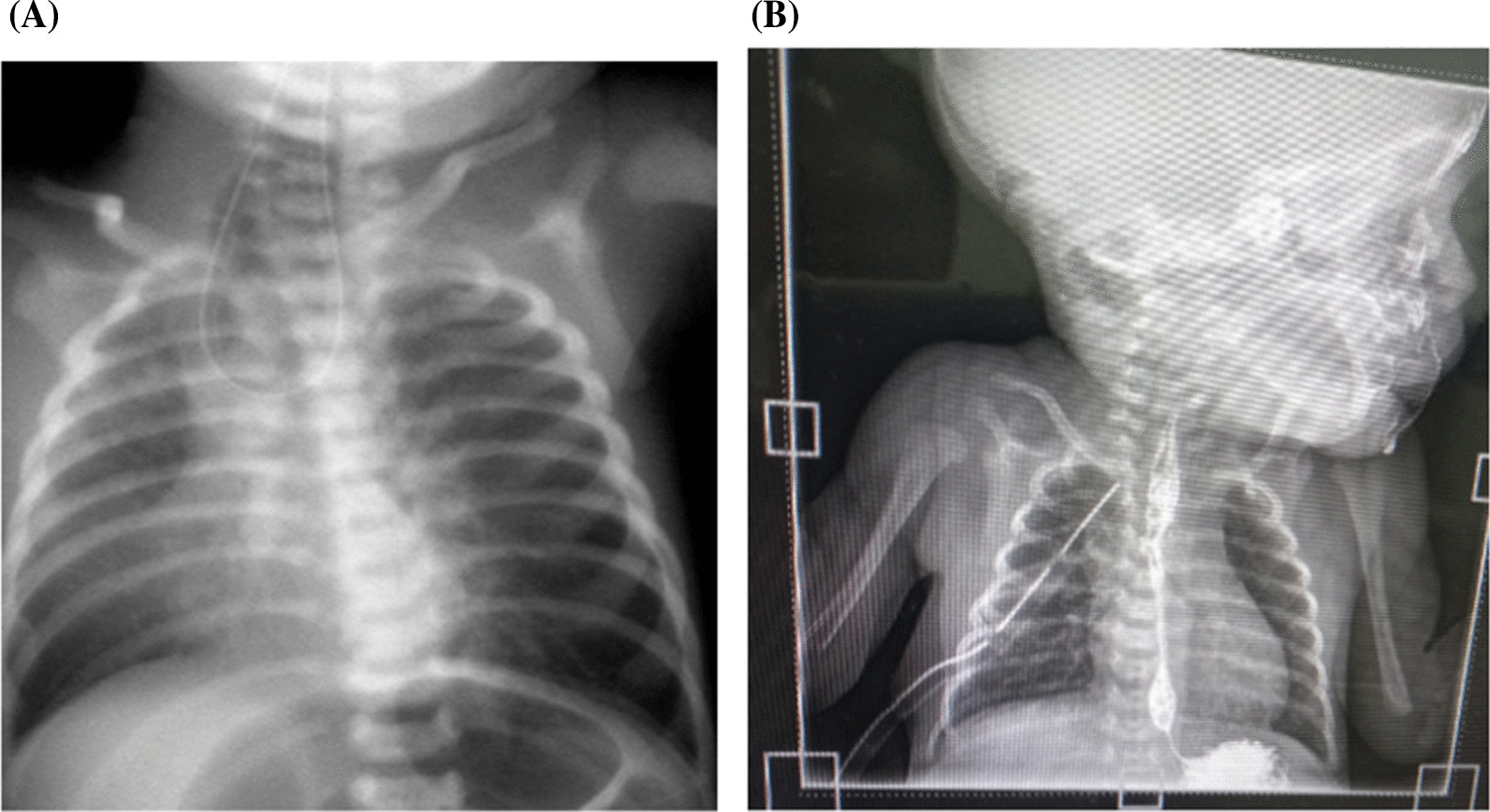
**A** Chest X-ray showing coiling of nasogastric tube. **B** Chest X-ray showing healed anastomosis

The investigations included a chest and abdominal X-ray, complete blood count, echocardiogram, and abdominal–pelvic ultrasound. Chest and abdominal X-ray showed proximal coiling of the nasogastric tube and gastric bubble features suggestive of complete esophageal atresia with associated distal tracheal esophageal fistula (Fig. 2A). The CBC showed thrombocytopenia. Bed side echocardiogram revealed moderate patent ductus arteriosus (PDA) with a left-to-right shunt.

On the third day of life, the baby underwent a right thoracotomy, which was successful. Intraoperative findings included blind-ended proximal esophagus and distal esophagus communicating with trachea-type C tracheoesophageal fistula. Fistula ligation was done, and end-to-end esophageal anastomosis was done. NGT was secured by sutures near the nostril. Postoperatively, the baby was managed in the NICU, and respiratory support was provided with noninvasive positive pressure ventilation (NIPPV). TPN continued.

On the 10th postoperative day, a gastrografin swallow was done to see progress of the anastomotic site and healing nature. It revealed a healing anastomosis; no leakage was noted (Fig. 2B). He was initiated on expressed breast milk, gradually increasing daily, to reach the maximum daily requirement of 180 ml/kg/day.

On 13th postoperative day he developed fever. CRP was raised, and blood culture revealed candida species; his hemoglobin dropped to 8 g/dl. Intravenous fluconazole was initiated and he was given a blood transfusion. The respiratory condition had improved significantly. The fever resolved in 3 days. He thereafter completed the course of fluconazole. Slow and graduated amount of breast milk was initially provided via NGT and cup feed, then direct breastfeeding was initiated. He was off ventilatory support and afterwards started to gain weight, so he was discharged at the age of 26 days. The colostomy continued to function and the parents were advised on the care of stoma and on return to clinic for follow-up.

## Discussion

TEF is one of the congenital birth defects that starts early in embryogenesis. It is an abnormal connection between the esophagus and the trachea postulated to develop due to the failure of separation or incomplete development of the caudal foregut [3]. The fistula tract derives from a branch of the embryonic lung bud that fails to undergo branching because of defective epithelial–mesenchyme interactions [6]. It has a male predominance and occurs mostly in late preterm and term babies [7]. Etiologies are unknown; however, theories have been postulated such as an esophageal occlusion and failure to recanalize, a spontaneous deviation of tracheoesophageal septum, or exposure to teratogens [8].

The patients we present are both term babies and were referred to the tertiary hospital for further management of TEF. Both were from mothers who were multipara, had no underlying diseases, and had negative serology. Both babies presented with respiratory distress and excessive secretion orally.

Anatomic classification is by E.C. Vogt, a radiologist. There are five subtypes (type A–E) of esophageal atresia in connection with the fistula, with type C being the most common [9]. The infants we present had type C on investigation.

Approximately 60% of neonates with EA and TEF have associated congenital anomalies such as cardiac anomalies, anal rectal malformation, renal anomalies, or limb anomalies [6, 9]. Cardiac anomalies are the most encountered and account for approximately one-third of all anomalies identified [8]. Gastrointestinal anomalies may include an imperforate anus, duodenal atresia, and malrotation, which make up one-fourth of the identified defects [10, 11]. As with the infants we present, both had patent ductus arteriosus (PDA), and the second case had an anorectal malformation.

Management of infants with TEF in our part of the world is still challenging. Antenatal-obstetric ultrasound can show polyhydramnions and absence of fetal stomach gas, however, both mothers in our cases had a normal scan. Due to delays in diagnosis, referral systems, and patient clinical condition at admission, the prognosis can be impacted. Cameron Haight performed the first successful esophageal atresia with a distal fistula in 1941 through a vertical incision; he entered the posterior mediastinum in the left chest, ligated the fistula, anastomosed the esophagus, and a rubber drain was left near the anastomosis [1]. The main treatment for TEF is surgery that can be performed with a right thoracotomy or bronchoscopy, and endoscopy is the primary therapeutic option [12]. Of the two cases we present, one had an earlier diagnosis and referral and had a good outcome postoperative. The infant with late diagnosis and referral succumbed, and because preoperative the baby was unwell, surgery was delayed due to initial stabilization; thus, there were postoperative complications.

Most neonates who undergo repair of esophageal atresia and tracheosephageal fistula have some degree of esophageal dysmotility [10]. Monthly follow up with our patient has been successful. No complains of reflux, difficulties in breathing, or feeding intolerance have been reported so far.

## Conclusion

Significant challenges in the repair for TEF exist in our region. The diagnosis of esophageal atresia requires a high degree of suspicion from the attending clinician. The delays in diagnosis, referral system, and patient clinical condition at admission can impact the prognosis. Therefore, early referral is imperative for timely management and excellent outcome. Mortality occurs due to development of aspiration pneumonia or anastomosis leak with development of pneumothorax. Once a diagnosis of esophageal atresia is established, measures should be taken to reduce the risk of aspiration and to support the airway. Preparations should be done for surgical correction as early as possible, especially when patient is stable.

## Data Availability

Not applicable.

## Abbreviations

EA: Esophageal atresia
CBC: Complete blood count
CRP: C-reactive protein
GA: Gestational age
HIV: Human immunodeficiency virus
LMIC: Lower middle income countries
NICU: Neonatal intensive care unit
NIPPV: Noninvasive positive pressure ventilation
PDA: Patent ductus arteriosus
TEF: Tracheoesophageal fistula
TPN: Total parental nutrition
VDRL: Venereal disease research laboratory
